# Enamel Caries Detection and Diagnosis: An Analysis of Systematic Reviews

**DOI:** 10.1177/00220345211042795

**Published:** 2021-10-12

**Authors:** T. Walsh, R. Macey, D. Ricketts, A. Carrasco Labra, H. Worthington, A.J. Sutton, S. Freeman, A.M. Glenny, P. Riley, J. Clarkson, E. Cerullo

**Affiliations:** 1 Division of Dentistry, School of Medical Sciences, The University of Manchester, Manchester, UK; 2 Dundee Dental School, University of Dundee, Dundee, UK; 3 Department of Evidence Synthesis and Translation Research, Science and Research Institute, LLC, American Dental Association, Chicago, IL, USA; 4 Department of Oral and Craniofacial Health Science, School of Dentistry, University of North Carolina at Chapel Hill, Chapel Hill, NC, USA; 5 Department of Health Sciences, University of Leicester, Leicester, UK

**Keywords:** evidence-based dentistry, radiography, transillumination, fluorescence, statistics, sensitivity and specificity

## Abstract

Detection and diagnosis of caries—typically undertaken through a visual-tactile examination, often with supporting radiographic investigations—is commonly regarded as being broadly effective at detecting caries that has progressed into dentine and reached a threshold where restoration is necessary. With earlier detection comes an opportunity to stabilize disease or even remineralize the tooth surface, maximizing retention of tooth tissue and preventing a lifelong cycle of restoration. We undertook a formal comparative analysis of the diagnostic accuracy of different technologies to detect and inform the diagnosis of early caries using published Cochrane systematic reviews. Forming the basis of our comparative analysis were 5 Cochrane diagnostic test accuracy systematic reviews evaluating fluorescence, visual or visual-tactile classification systems, imaging, transillumination and optical coherence tomography, and electrical conductance or impedance technologies. Acceptable reference standards included histology, operative exploration, or enhanced visual assessment (with or without tooth separation) as appropriate. We conducted 2 analyses based on study design: a fully within-study, within-person analysis and a network meta-analysis based on direct and indirect comparisons. Nineteen studies provided data for the fully within-person analysis and 64 studies for the network meta-analysis. Of the 5 technologies evaluated, the greatest pairwise differences were observed in summary sensitivity points for imaging and all other technologies, but summary specificity points were broadly similar. For both analyses, the wide 95% prediction intervals indicated the uncertainty of future diagnostic accuracy across all technologies. The certainty of evidence was low, downgraded for study limitations, inconsistency, and indirectness. Summary estimates of diagnostic accuracy for most technologies indicate that the degree of certitude with which a decision is made regarding the presence or absence of disease may be enhanced with the use of such devices. However, given the broad prediction intervals, it is challenging to predict their accuracy in any future “real world” context.

## Introduction

Detection and diagnosis of caries are typically undertaken through visual-tactile examination by a general dental practitioner, often with supporting radiographic investigations, and this is commonly regarded as being broadly effective at detecting caries that has progressed into dentine and reached a threshold where restoration is necessary (Kidd and Fejerskov 2004). Active caries presenting at earlier levels into tooth enamel and outer aspects of dentine has the potential to be stabilized or even reversed, whereas the progression of lesions deeper into the dentine and pulp of the tooth will typically require restoration, particularly if the surface of the tooth has broken down (cavitated). The detection of caries earlier in the disease continuum offers the opportunity for nonsurgical treatment aimed at remineralization of the tooth surface, with the goal of maximizing retention of tooth tissue and preventing the patient from entering a lifelong cycle of restoration (Pitts et al. 2017). A variety of treatment options are available at different thresholds of disease. Initially, advising improved self-care with age-appropriate concentration fluoride toothpaste, reduction of sugar consumption, or topical fluoride supplements may be recommended or applied by a dental professional (Kidd and Fejerskov 2016). Minimally invasive nonoperative treatments, such as sealing the affected surface of the tooth or “infiltrating” the demineralized tissue with resins, may be undertaken for initial caries, although the certainty of the evidence for the effectiveness of such interventions varies according to tooth surface (Urquhart et al. 2019). Selective or stepwise caries removal and restoration may be necessary for more extensive lesions (Ismail et al. 2013).

The need to clinically assess the severity and activity of dental caries to define treatment has influenced the development and refinement of diagnostic technologies that purport to discriminate between sound and diseased tooth tissue. Systematic reviews of caries diagnosis have largely focused on a single or small number of technologies, leaving clinicians, patients, and other stakeholders with the burden of processing large bodies of evidence across technologies and multiple review reports to inform their decisions (e.g., Bader et al. 2002; Gimenez et al. 2013; Gimenez et al. 2015; Schwendicke et al. 2015). Furthermore, current recommended methodologies have not always been adopted (Macaskill et al. 2010; Schünemann et al. 2020a, 2020b). For stakeholders, a comprehensive synthesis that provides robust information on the comparative diagnostic accuracy of several technologies is required for clinical decision making but is currently lacking.

To inform the detection and diagnosis of early caries, we recently authored a suite of Cochrane diagnostic test accuracy (DTA) reviews of 1) fluorescence, 2) visual or visual-tactile examination according to detailed criteria, 3) radiographic imaging and cone beam computed tomography (CBCT), 4) optical coherence tomography and transillumination, and 5) electrical conductance or impedance (Macey et al. 2020; Macey, Walsh, Riley, Glenny, Worthington, Clarkson, et al. 2021; Macey, Walsh, Riley, Glenny, Worthington, O’Malley, et al. 2021; Macey, Walsh, Riley, Hogan, et al. 2021; Walsh et al. 2021). These were published as separate reviews, so only naïve comparisons of the technologies could be made. The primary aim of this research was to undertake a formal comparative analysis of the diagnostic accuracy of these technologies to provide a firm foundation on which to base clinical decision making, clinical guidelines, and policy. Our objectives were to undertake a statistically robust comparative evaluation of the DTA of the aforementioned technologies and to assess the certainty of the evidence with the GRADE approach (Schünemann et al. 2020a, 2020b).

## Methods

We identified 5 Cochrane DTA systematic reviews meeting the following criteria (Macey et al. 2020; Macey, Walsh, Riley, Glenny, Worthington, Clarkson, et al. 2021; Macey, Walsh, Riley, Glenny, Worthington, O’Malley, et al. 2021; Macey, Walsh, Riley, Hogan, et al. 2021; Walsh et al. 2021).

*Participants*: children, adolescents, and adults seemingly asymptomatic for caries.
*Types of studies*: In vivo (intraoral) and in vitro (extracted teeth) studies with a single set of inclusion criteria that compared a diagnostic test with a reference standard or case-control–type accuracy studies where different sets of criteria were used to recruit those with or without the target condition. Studies were excluded if numbers of true and false positives and negatives could not be obtained.
*Target condition*: Coronal caries at initial stage decay, defined as initial or incipient caries or noncavitated lesions, including lesions adjacent to restorations (Young et al. 2015). Specifically, there is a detectable change in enamel that is not thought to have progressed into dentine at the point of recruitment on occlusal, approximal, or smooth surfaces. This target condition was chosen as earlier detection provides clinicians with an opportunity to stabilize lesion progression or even remineralize the tooth surface.
*Index tests*: 1) fluorescence at red, blue, and green wavelengths that included Diagnodent, MidWest, VistaProof, SoproLife, and quantitative light-induced fluorescence devices; 2) visual or visual-tactile classification systems, principally the International Caries Detection and Assessment System (ICDAS), the Ekstrand-Ricketts-Kidd system, and the Nyvad system; 3) imaging (analog or digital radiographs, CBCT); 4) transillumination—including fiber-optic transillumination, digital fiber-optic transillumination, and near-infrared transillumination—and optical coherence tomography; and 5) electrical conductance or impedance.
*Reference standard*: histology. When this was not available or appropriate, operative exploration and enhanced visual assessment (with or without tooth separation) were considered acceptable alternatives.

For the proposed comparative analysis, 1) within-study, within-person or 2) within-study, between-person randomized studies comprising all technologies of interest would be the optimal designs. A direct within-study, within-person comparison is made when an individual undergoes multiple index tests within the same study, which are then verified by a reference standard. A within-study, between-person comparison is made when individuals within a study are allocated, preferably randomly, to receive different index tests and are then verified by a reference standard. Comparative analyses based on within-study, within-person or within-study, between-person designs are generally favored over those based on between-study comparisons, as confounding is reduced and bias minimized with the former designs. Empirical evidence suggests that inferences from within-study analyses and between-study analyses can differ and that within-person or within-study, between-person study designs are preferred when the key consideration is comparative accuracy (Takwoingi et al. 2013).

Initial consideration of the Cochrane reviews indicated that there were no studies meeting those criteria. Therefore, we planned to include all studies that reported the evaluation of ≥2 technologies for the same individual within a study, verified by a suitable reference standard, and to consider the within-study, within-person and between-person evidence separately. Where a primary study provided >1 data set per technology (e.g., analog and digital radiographs) to minimize dependency of data within an analysis, the data set with the largest volume of data was included in the analysis. This decision was justified on the basis that for each systematic review, no differences in accuracy estimates were typically observed within the individual technologies.

Screening of the studies for inclusion in this comparative review was done independently and in duplicate. We used the QUADAS-2 assessments from the original Cochrane reviews as an indication of methodological quality (Whiting et al. 2011), and we used GRADE to assess the certainty of the evidence (Schünemann et al. 2020a, 2020b).

### Statistical Analysis

We conducted 2 separate analyses based on study design. First, we conducted a fully within-study, within-person analysis and included studies that directly evaluated ≥3 of the same multiple index tests. The second analysis took a network meta-analysis (NMA) approach and was based on direct and indirect test comparisons where at least 2 index tests had been directly compared. One important downside of this latter approach is that any gains in precision resulting from the increased number of available studies are offset by potentially biasing the estimates of the differences between technologies, due to systematic differences in the studies that evaluated the different technologies.

While between-study heterogeneity was often substantial in the systematic reviews, meta-regressions considering the impact of dentition, tooth surface, or reference standard/study design could not explain this heterogeneity; therefore, our approach was an analysis of all studies (without including these covariates). We used summary receiver operating characteristic plots to illustrate the sensitivity and specificity points for each study. Summary sensitivity and specificity points were plotted to indicate the summary operating points for the different technologies, with 95% credible intervals and prediction regions, the latter to indicate the region within which the true sensitivity and specificity of a future study can be expected to lie (Harbord et al. 2007). For the fully within-person analysis, linked receiver operating characteristic plots were used to illustrate the change in accuracy within a study between the technologies. Pairwise differences in summary sensitivity and specificity points with 95% credible intervals (CrIs) were used to evaluate differences in comparative accuracy. We also carried out an exploratory analysis for fluorescence, stratifying the data by grouping multiple thresholds and conducting a series of stratified bivariate analyses (Reitsma et al. 2005; Roberts et al. 2015).

For the first analysis, a fully within-study, within-person analysis, we used a Bayesian version of the model from Hoyer and Kuss (2016), extended to 3 tests. This model is an extension to that proposed in the *Cochrane Handbook for Systematic Reviews of Diagnostic Test Accuracy* (Macaskill et al. 2010) and allows between-study correlation parameters between tests to be explored. The second analysis used a NMA model (Nyaga et al. 2016). We coded all models in Stan using cmdstanr (Carpenter et al. 2017; Češnovar et al. 2021). We compared fit between models using cross-validation (Vehtari et al. 2017). We ran all models until all of the split R-hat statistics for all parameters were <1.05 and parameters had at least 100 effective samples, and we checked all posterior distributions and trace plots. Prior distributions are documented in the Appendix, and data and code are available from https://github.com/CerulloE1996/Walsh-et-al-analysis.

### Analysis 1: Fully Within-Person Comparison

The first model fitted (M1) had the most complex between-study model structure and estimated correlations between and within technologies (i.e., between sensitivities and specificities). The second model (M2) was a simpler version of M1, with all of the between-test correlations set to zero, equivalent to fitting separate bivariate models for each technology (Reitsma et al. 2005). Finally, the simplest model (M3) fitted a single shared correlation parameter and shared between-study heterogeneity for all 3 variance-covariance matrices.

### Analysis 2: Comparison Based on All Studies With at Least 2 Index Tests (NMA)

The first model fitted (M1-NMA) estimated separate correlation and variance parameters for all 5 technologies. The second model (M2-NMA) was a simpler model, which assumed the same correlation and variances across all technologies.

## Results

The Cochrane reviews comprised 158 studies, of which 68 evaluated within-study, within-person comparisons of ≥2 technologies (Appendix Figs. 1 and 2, Appendix Table 1). No studies reported the evaluation of CBCT with any other technology for the same individual within a study.

**Table 1.** Summary Sensitivity and Specificity Operating Points With 95% CrI and 95% PrI.

	Analysis 1^a^		Analysis 2^b^	
	Sensitivity (95% CrI) [95% PrI]	Specificity (95% CrI) [95% PrI]	Sensitivity (95% CrI) [95% PrI]	Specificity (95% CrI) [95% PrI]
Fluorescence	0.71 (0.59, 0.81) [0.10, 0.98]	0.88 (0.78, 0.94) [0.18, 1.00]	0.76 (0.68, 0.82) [0.20, 0.97]	0.83 (0.75, 0.89) [0.23, 0.99]
Imaging	0.48 (0.35, 0.62) [0.04, 0.98]	0.92 (0.84, 0.96) [0.23, 1.00]	0.50 (0.40, 0.59) [0.07, 0.92]	0.89 (0.83, 0.93) [0.31, 0.99]
Visual classification	0.82 (0.73, 0.89) [0.17, 0.99]	0.85 (0.74, 0.93) [0.13, 1.00]	0.83 (0.77, 0.87) [0.28, 0.98]	0.81 (0.73, 0.87) [0.19, 0.99]
Electrical conductance or impedance	NA	NA	0.83 (0.66, 0.92) [0.24, 0.99]	0.72 (0.44, 0.89) [0.12, 0.98]
Transillumination OCT	NA	NA	0.76 (0.63, 0.86) [0.20, 0.98]	0.82 (0.68, 0.91) [0.21, 0.99]

Parentheses indicate 95% credible intervals, and brackets indicate 95% prediction intervals.

NA, not applicable; OCT, optical coherence tomography.

a Fully within-person comparison (19 studies, 2,849 tooth sites or surfaces).

b Comparison based on all studies that evaluated at least 2 technologies (64 studies, 24,567 tooth sites or surfaces).

Only 1 study was judged as low risk of bias across all QUADAS-2 risk-of-bias domains, but 15 studies were judged as low concern for all applicability domains. Low risk-of-bias judgments were attributed to 7 studies for patient selection domain, 46 studies for the reference standard domain, and 56 studies for the flow-and-timing domain. Risk-of-bias judgments varied by technology. Low concern for applicability judgments was attributed to 23 studies for the patient selection domain, varied across the index test domain, and attributed to 65 studies for the reference standard domain (Appendix Table 2).

**Table 2.** Summary of Findings and GRADE Assessment.

Question	What is the comparative diagnostic accuracy of technologies to detect and inform the diagnosis of early dental caries?					
Population	Children or adults presenting asymptomatically or who are suspected of having enamel caries (clinical studies); extracted teeth (in vitro studies). Studies that intentionally included dentine and frank cavitations were excluded.					
Index test	Visual classification (ICDAS, ERK, other), fluorescence-based devices (red, blue, and green wavelengths), imaging (analog and digital radiographs), electrical conductance or impedance, transillumination and OCT.					
Target condition	Dental caries, at the threshold of caries in enamel.					
Reference standard	Histology, excavation, enhanced visual examination with or without radiographs.					
Action	Early caries was chosen as the target condition as an appropriate time for clinical intervention when remedial preventive action can be taken to arrest or reverse the decay and potentially prevent restorations.					
Diagnostic stage	Aimed at the general dental practitioner assessing patients for early-stage caries.					
Quantity of evidence	64 studies providing data for meta-analysis,^a^ 24,567 tooth surfaces (70% prevalence caries at enamel threshold).					
		Findings				
		Visual	Fluorescence	Imaging	Electrical Conductance	Transillumination OCT
Sensitivity (95% CrI) [95% PrI]		0.83 (0.77, 0.87) [0.28, 0.98]	0.76 (0.68, 0.82) [0.20, 0.97]	0.50 (0.40, 0.59) [0.07, 0.92]	0.83 (0.66, 0.92) [0.24, 0.99]	0.76 (0.63, 0.86) [0.20, 0.98]
Specificity (95% CrI) [95% PrI]		0.81 (0.73, 0.87) [0.19, 0.99]	0.83 (0.75, 0.89) [0.23, 0.99]	0.89 (0.83, 0.93) [0.31, 0.99]	0.72 (0.44, 0.89) [0.12, 0.98]	0.82 (0.68, 0.91) [0.21, 0.99]
	Effect per 1,000 Tooth Surfaces (95% CI) at a Prevalence of 28%					COE
True positives	232 (216, 244)	213 (190, 2330)	140 (112, 165)	232 (185, 258)	213 (176, 241)	Low^b^
False negatives (missed cases)	48 (36, 64)	67 (50, 90)	140 (115, 168)	48 (22, 95)	67 (39, 104)	
True negatives	583 (526, 626)	598 (540, 641)	641 (598, 670)	518 (317, 641)	590 (490, 655)	Low^b^
False positives (potential for overdiagnosis)	137 (94, 194)	122 (79, 180)	79 (50, 122)	202 (79, 403)	130 (65, 230)	

An illustrative prevalence of 28% was taken from the UK Adult Dental Health Survey (Steele and O’Sullivan 2011) indicating the prevalence of primary or secondary caries into coronal dentine.

95% CrI, 95% credible interval; 95% PrI, 95% prediction interval; COE, certainty of the evidence (GRADE); ERK, Ekstrand-Ricketts-Kidd; ICDAS, International Caries Detection and Assessment System; OCT, optical coherence tomography.

a Nineteen studies providing data from the fully within-person meta-analysis.

b We graded the certainty of evidence as low and downgraded 2 levels in total due to risk of bias (primarily from nonconsecutive or nonrandom selection of observations), inconsistency (unexplained heterogeneity reflected in the large 95% prediction regions), and indirectness (a comparatively large proportion of studies evaluated the accuracy of the technologies on extracted teeth).

Details regarding the characteristics of studies and rationale for the QUADAS-2 assessments are available from the Cochrane reviews.

### Analysis 1: Fully Within-Person Comparison

While the data sets used in analysis 1 offered the benefit of minimizing bias due to confounding, the analysis was limited in that data from a small subset of studies were included in the meta-analysis. The most commonly occurring configuration of ≥3 technologies within a study was fluorescence, visual classification, and dental imaging (analog or digital radiographs), as reported in 19 studies (19 data sets, 2,849 tooth sites or surfaces, 66% enamel caries prevalence). None of the studies in this analysis presented the data in fully “paired” form—that is, 2 × 4 tables of the results of each index test cross-classified by cases and noncases.

We were unable to carry out this analysis on any other combination of ≥3 technologies due to an insufficient number of studies (Appendix Fig. 2).

**Figure 1.**
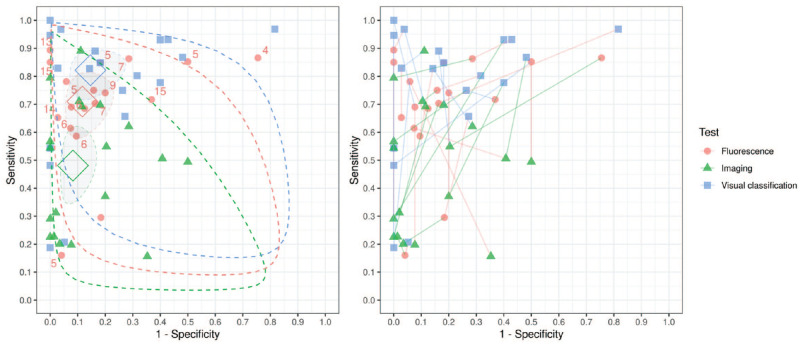
Sensitivities and specificities based on data from 17 studies that evaluated 3 index tests and 2 studies that evaluated 4 index tests (19 studies reporting within-person comparisons and 2,849 tooth sites or surfaces). Summary receiver operating characteristic plots illustrate the sensitivity and specificity points for each study. (Left) Hollow tilted squares indicate the summary points for each technology; shaded areas indicate 95% credible regions; dotted lines indicate 95% prediction regions; numbers indicate test-positive thresholds for fluorescence studies. (Right) A linked plot illustrates the within-study change in accuracy between the technologies by connecting the 3 results for each study with a line.

Of the 3 proposed models (M1, M2, and M3), there was no evidence of a difference in model fit, so we used the simplest model, M3, for inference. Accuracy estimates varied within and between technologies (Fig. 1, see also Table 1).

The reasonably narrow confidence regions reflect the volume of data in the analysis, whereas the broad prediction regions indicate the large variability of results among studies and imply uncertainty of the diagnostic accuracy of each technology in any particular context.

Visual classification and fluorescence outperformed radiographic imaging in terms of sensitivity. Pairwise differences in sensitivity between technologies were as follows:

- Sensitivity of fluorescence minus sensitivity of imaging: 0.23 (95% CrI, 0.05, 0.39)
- Sensitivity of imaging minus sensitivity of visual classification: −0.34 (95% CrI, −0.49, −0.17)
- Sensitivity of fluorescence minus sensitivity of visual classification: −0.11 (95% CrI, −0.25, 0.03)

Specificity estimates were broadly similar. Pairwise differences in specificity between technologies were as follows:

- Specificity of fluorescence minus specificity of imaging: −0.03 (95% CrI, −0.14, 0.06)
- Specificity of imaging minus specificity of visual classification: 0.06 (95% CrI, −0.04, 0.19)
- Specificity of fluorescence minus specificity of visual classification: −0.03 (95% CrI, −0.09, 0.16)

The intrastudy correlation coefficient—a measure of the proportion of variability in the sensitivity or specificity (on the logistic scale) that is accounted for by the between-study variability—was 0.41 (95% CrI, 0.21, 0.59) and 0.47 (95% CrI, 0.23, 0.66), respectively. This suggests that the variability was roughly evenly split between within- and between-study variability.

### Analysis 2: Comparison Based on All Studies With at Least 2 Index Tests

Sixty-four studies (24,567 tooth sites, 70% prevalence of enamel caries) providing a within-study, within-person comparison with at least 1 other technology were included in the meta-analysis. Data were available for all 5 technologies of interest.

**Figure 2.**
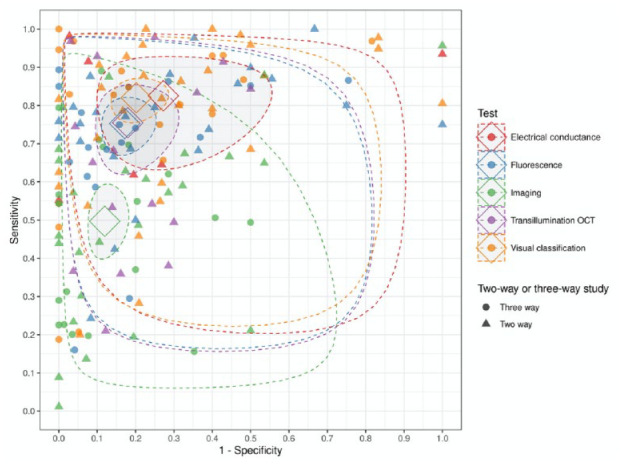
Summary receiver operating characteristic plot illustrates the sensitivity and specificity points for each study. Hollow tilted squares indicate the summary points for each technology; shaded areas with dotted boundaries indicate 95% credible regions; dotted boundaries (no shading) indicate 95% prediction regions. Data are based on the network meta-analysis comparison of 64 studies (66 data sets with 24,567 tooth sites or surfaces). OCT, optical coherence tomography.

Of the 2 proposed models, M1-NMA and M2-NMA, we observed no evidence of a difference in model fit, and so we used the simpler model, M2-NMA, for inference. Variation in accuracy estimates within and between technologies could be observed (Fig. 2, see also Table 1).

The larger 95% confidence regions for electrical conductance or impedance and transillumination can be considered reflective of the smaller volume of available data. However, the 95% prediction regions are broad for all technologies, indicating the unexplained heterogeneity and uncertainty regarding diagnostic accuracy in any given context.

**Figure 3.**
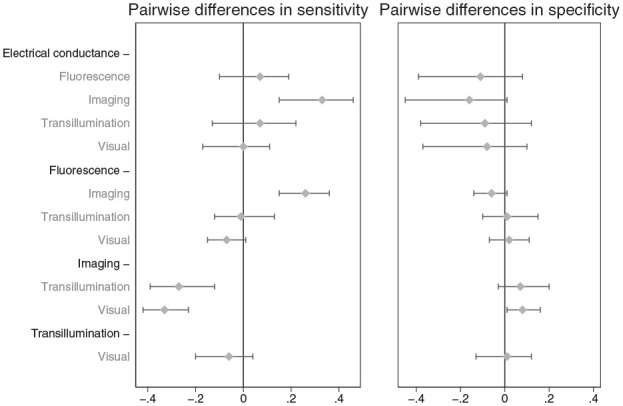
Pairwise differences in summary sensitivity and specificity points with 95% credible intervals were used to evaluate differences in comparative accuracy.

The summary sensitivity estimates were highest for electrical conductance or impedance and visual classification, followed by fluorescence and transillumination, with radiographic imaging the least sensitive and significantly lower than all other technologies. The summary specificity estimates were similar across the different technologies, however (Table 1). The wide 95% prediction intervals are indicative as to the uncertainty of future diagnostic accuracy for all technologies. Analysis of pairwise differences in sensitivity confirmed that the diagnostic accuracy of dental imaging was poorer than the other technologies studied but that specificity was similar (Fig. 3).

A stratified bivariate analysis for fluorescence-based technologies reporting on a continuous scale and grouping studies with similar thresholds indicated, somewhat unexpectedly, no discernible association between test positivity threshold and accuracy estimates (Appendix Fig. 3). Within any given study, accuracy must increase with test positivity threshold. Since each study reported accuracy at a single threshold, it is likely that other between-study factors are masking the association between threshold and accuracy.

Estimates for the 3 technologies evaluated in analyses 1 and 2 were consistent. Based on analysis 2, we graded the certainty of evidence as low and downgraded 2 levels due to risk of bias (primarily from nonconsecutive or nonrandom selection of observations), inconsistency (unexplained heterogeneity reflected in the large 95% prediction regions), and indirectness (a comparatively large proportion of studies evaluated the accuracy of the technologies on extracted teeth; Table 2).

## Discussion

We conducted a comparative analysis of a suite of 5 Cochrane DTA systematic reviews on the application of technologies to detect and inform the diagnosis of initial caries.

### Summary of Main Findings

Our initial approach to determining comparative accuracy was to include only studies reporting the direct analysis of multiple technologies in a single model that allowed direct comparisons. While the use of fully within-person study designs is advantageous in terms of minimizing the potential for bias due to confounding, we observed important disadvantages to this approach: the number of eligible studies was reduced from 158 to 19; there were 5 broad categories of technologies under evaluation, and no single study evaluated all 5; and the comparative analysis was driven by the pattern and availability of data rather than clinical interest. Taking an NMA approach to the comparative analysis of studies that evaluated >1 technology meant that more studies could be included in the meta-analysis (64 studies vs. 19). As a result, we were able to evaluate all the technologies of interest with a greater degree of precision but with a caveat: estimates of the differences between tests could be biased due to systematic differences among the studies that evaluated the different technologies.

The diagnostic accuracy of the tests was similar for the fully within-person analysis and the NMA, which suggests confidence in the robustness of the results. In terms of sensitivity, the comparative performance of the technologies was similar, with the exception of radiographic imaging, which exhibited the poorest performance, reflective of the findings of Gimenez et al. (2021) for caries at all levels; the summary estimates of specificity were similar across all technologies. However, with both methodological approaches to analysis, we observed considerable variation in the accuracy estimates from the primary studies, as reflected in the 95% prediction regions. In each systematic review, we formally investigated prespecified potential sources of heterogeneity in terms of tooth surface, dentition, reference standard, prevalence of caries into dentine undetected at the point of recruitment, and clinical or laboratory study through meta-regression and found that there was typically no difference in accuracy estimates. Therefore, it is unlikely that these factors are drivers for the differences in accuracy observed across the technologies, although differences in study design, conduct, and analysis potentially contribute to the variability of the observed results (i.e., heterogeneity). Our research could be extended to explore the potential effects of covariates through the use of multiple meta-regression coefficients. Such an approach would have to assume that there is a convincing clinical rationale that underpins any additional analyses. Given the broad prediction intervals for all technologies, it is currently challenging to predict their diagnostic accuracy in any particular “real world” context.

The original QUADAS-2 assessment indicated that there were shortcomings in the body of evidence. These were partly due to unavoidable complexities in study design and conduct arising from issues such as the use of an imperfect reference standard and data-driven thresholds for classification of disease when device-specific manufacturer guidance is not available. Other identified limitations would be easier to rectify in future studies, such as nonconsecutive or nonrandom recruitment or a lack of blinding to results when multiple technologies are employed.

We identified issues of indirectness due to the dominance of studies where both the technology under evaluation and the reference standard were conducted on extracted teeth. A supplementary sensitivity analysis that included only in vivo studies was not feasible due to the complexity of the statistical methods and the small number of studies available.

For an analysis of existing systematic reviews, we elected to retain the original categorizations of technologies as presented in the peer-reviewed protocol and resultant reviews. We acknowledge that alternative categorizations may be of interest, and so the data and statistical methods have been made publicly available.

Preclinical studies are an important part of the development of diagnostic tests; however, the generalizability of results can be called into question when the intended use is on teeth in situ, with the accompanying difficulties of access to the oral cavity, plaque, tooth staining, and patient discomfort.

Despite the large volume of data, we judged the certainty of the evidence to be low.

### Implications for Research

Useful additions to the evidence base would include within-person comparative studies carried out in a clinical setting that focus on minimizing bias arising from the use of imperfect reference standards and that report the results across all levels of disease severity. The design and conduct of clinical studies (in vivo) are more complex than for laboratory studies on extracted teeth (in vitro), and this is largely reflected in the existing evidence base. When inferences from in vivo and in vitro studies are considered, there is often an implicit trade-off between risk of bias and applicability, specifically with respect to the available reference standard and the use of the technology in practice. A reference standard from an in vivo study is less likely to correctly classify early caries than a reference standard of histology from an in vitro study. The conduct or interpretation of the technology under evaluation in an in vitro study on extracted teeth could elicit some concern regarding applicability, as it may not be reflective of how the technology would be used in routine practice, but there would be low applicability concerns for in vivo studies in this regard. To maximize applicability to clinical practice, one possible study design, albeit logistically difficult to conduct, is a clinical study where the technology is applied to teeth in situ that are due to be extracted, thus permitting the use of histology as a reference standard. Even with this study design, consideration should be given to the broader external validity of such studies, which are most likely to recruit adolescents or younger adults, who may have a lower prevalence of disease than an adult population and therefore may not be representative of the wider population. For secondary research, there is the option of conducting a sensitivity analysis with only in vivo studies or limiting eligibility to in vivo studies. This would result in a large volume of research data being discarded, but more important, the certainty of the evidence would still be affected by study limitations from the use of an imperfect reference standard.

Additionally, future comparative DTA studies should be comprehensively reported, including tables of results of the index tests cross-classified among cases and noncases to fully incorporate the data dependency, and anonymized individual patient data should be made available. In this research, few studies provided data at multiple positivity thresholds, and so we were unable to explicitly model the effects at different thresholds.

Last, randomized studies considering health outcomes and cost-effectiveness according to different diagnostic strategies, including early versus late detection and diagnosis, should be undertaken to broaden the current evidence base and inform clinical guidelines.

### Implications for Clinical Practice

Diagnostic tests should always be contextualized in the clinical pathway, acknowledging that they are intrinsically connected within the continuum of disease treatment and management. Thus, DTA estimates are just a surrogate and serve as preliminary data for more appropriate and directly linked evidence that focuses on patient-important outcomes (benefits and harms of the test strategy under evaluation), resource utilization, and impact on equity in the health system. In the absence of direct evidence on patient-important outcomes, clinicians, patients, and policy makers may still take advantage of DTA estimates by hypothesizing the downstream consequences associated with true-positive, true-negative, false-positive, and false-negative results and their magnitude (Schünemann et al. 2019).

Given the proportions of false-negative and false-positive results, it may seem that there is little benefit in supplementing the visual or visual-tactile method of caries detection with the use of more novel technologies. Typically, the majority of primary care practitioners do not use a comprehensive robust visual/visual-tactile classification system, such as ICDAS or Ekstrand-Ricketts-Kidd, for caries detection; consequently, there is a risk of failing to detect early lesions in routine clinical practice. In such instances, the use of these novel technologies may be beneficial. However, where a practitioner employs a robust and detailed ICDAS examination for every patient, then the use of these technologies may confer little additional benefit. The objective assessment provided by some of the technologies may be of benefit in monitoring lesions detected early and managed preventatively and overcoming issues in the use of visual classification systems, such as variability of application in a general practice setting, individual variation in the interpretation of the severity of carious lesions, and subjective recall over time.

While summary estimates of diagnostic accuracy indicate that the degree of certitude with which a decision is made regarding the presence or absence of early disease may be enhanced with the use of some of the technologies evaluated, it is challenging to predict their diagnostic accuracy in any future “real world” context given the broad prediction intervals.

## Author Contributions

T. Walsh, R. Macey, contributed to conception, design, data acquisition, analysis, or interpretation, drafted and critically revised the manuscript; D. Ricketts, contributed to conception, design, and data interpretation, drafted and critically revised the manuscript; A. Carrasco Labra, contributed to data interpretation, drafted and critically revised the manuscript; H. Worthington, A.M. Glenny, P. Riley, contributed to conception, design, data acquisition, and interpretation, critically revised the manuscript; A.J. Sutton, contributed to conception, design, data analysis or interpretation, drafted and critically revised the manuscript; S. Freeman, E. Cerullo, contributed to conception, design, data analysis, or interpretation, drafted and critically revised the manuscript; J. Clarkson, contributed to conception, design, and data interpretation, critically revised the manuscript. All authors gave final approval and agree to be accountable for all aspects of the work.

## Supplemental Material

sj-pdf-1-jdr-10.1177_00220345211042795 – Supplemental material for Enamel Caries Detection and Diagnosis: An Analysis of Systematic Reviews Click here for additional data file. Supplemental material, sj-pdf-1-jdr-10.1177_00220345211042795 for Enamel Caries Detection and Diagnosis: An Analysis of Systematic Reviews by T. Walsh, R. Macey, D. Ricketts, A. Carrasco Labra, H. Worthington, A.J. Sutton, S. Freeman, A.M. Glenny, P. Riley, J. Clarkson and E. Cerullo in Journal of Dental Research

## References

[bibr1-00220345211042795] Bader JD Shugars DA Bonito AJ . 2002. A systematic review of the performance of methods for identifying carious lesions. J Public Health Dent. 62(4):201–213. 12474624 10.1111/j.1752-7325.2002.tb03446.x

[bibr2-00220345211042795] Carpenter B Gelman A Hoffman MD Lee D Goodrich B Betancourt M Brubaker M Guo J Li P Riddell A . 2017. Stan: a probabilistic programming language. J Stat Softw. 76(1):1–32. 10.18637/jss.v076.i01 PMC9788645 36568334

[bibr3-00220345211042795] Češnovar J Bales B Morris M Popov M Lawrence M . 2021. Cmdstanr: a lightweight interface to stan for R users. R package version 0.3.0.

[bibr4-00220345211042795] Gimenez T Braga M Raggio D Deery C Ricketts D Mendes F . 2013. Fluorescence-based methods for detecting caries lesions: systematic review, meta-analysis and sources of heterogeneity. PLoS One. 8(4):e60421. 23593215 10.1371/journal.pone.0060421 PMC3617206

[bibr5-00220345211042795] Gimenez T Piovesan C Braga MM Raggio DP Deery C Ricketts DN Ekstrand KR Mendes FM . 2015. Visual inspection for caries detection: a systematic review and meta-analysis. J Dent Res. 94(7):895–904. 25994176 10.1177/0022034515586763

[bibr6-00220345211042795] Gimenez T Tedesco T Janoian F Braga M Raggio D Deery C Ricketts D Ekstrand K Mendes F . 2021. What is the most accurate method for detecting caries lesions? A systematic review. Community Dent Oral Epidemiol. 49(3):216–224. 33847007 10.1111/cdoe.12641

[bibr7-00220345211042795] Harbord RM Deeks JJ Egger M Whiting P Sterne JA . 2007. A unification of models for meta-analysis of diagnostic accuracy studies. Biostatistics. 8(2):239–251. 16698768 10.1093/biostatistics/kxl004

[bibr8-00220345211042795] Hoyer A Kuss O . 2016. Meta-analysis for the comparison of two diagnostic tests to a common gold standard: a generalized linear mixed model approach. Stat Methods Med Res. 27(5):1410–1421. 27487844 10.1177/0962280216661587

[bibr9-00220345211042795] Ismail AI Tellez M Pitts NB Ekstrand KR Ricketts D Longbottom C Eggertsson H Deery C Fisher J Young DA , et al. 2013. Caries management pathways preserve dental tissues and promote oral health. Community Dent Oral Epidemiol. 41(1):e12–e40. 24916676 10.1111/cdoe.12024

[bibr10-00220345211042795] Kidd E Fejerskov O . 2016. Essentials of dental caries. New York (NY): Oxford University Press.

[bibr11-00220345211042795] Kidd EAM Fejerskov O . 2004. What constitutes dental caries? Histopathology of carious enamel and dentin related to the action of cariogenic biofilms. J Dent Res. 83(Spec No C):C35–C38. doi:10.1177/154405910408301s07 15286119

[bibr12-00220345211042795] Macaskill P Gatsonis C Deeks JJ Harbord RM Takwoingi Y . 2010. Analysing and presenting results. In: Deeks JJ Bossuyt PM Gatsonis C , editors. Cochrane handbook for systematic reviews of diagnostic test accuracy version 1.0. London (UK): The Cochrane Collaboration. Chap. 10.

[bibr13-00220345211042795] Macey R Walsh T Riley P Glenny AM Worthington HV Clarkson JE Ricketts D . 2021. Electrical conductance for the detection of dental caries. Cochrane Database Syst Rev. 3:CD014547. 10.1002/14651858.CD014547 PMC8406820 33724442

[bibr14-00220345211042795] Macey R Walsh T Riley P Glenny AM Worthington HV Fee PA Clarkson JE Ricketts D . 2020. Fluorescence devices for the detection of dental caries. Cochrane Database Syst Rev. 12:CD013811. 10.1002/14651858.CD013811 PMC8677328 33319353

[bibr15-00220345211042795] Macey R Walsh T Riley P Glenny AM Worthington HV O’Malley L Clarkson JE Ricketts D . 2021. Visual or visual-tactile examination to detect and inform the diagnosis of enamel caries. Cochrane Database Syst Rev. 6:CD014546. 10.1002/14651858.CD014546 PMC8428329 34124773

[bibr16-00220345211042795] Macey R Walsh T Riley P Hogan R Glenny AM Worthington HV Clarkson JE Ricketts D . 2021. Transillumination and optical coherence tomography for the detection and diagnosis of enamel caries. Cochrane Database Syst Rev. 1:CD013855. 10.1002/14651858.CD013855 PMC8487162 33502759

[bibr17-00220345211042795] Nyaga VN Aerts M Arbyn M . 2016. Anova model for network meta-analysis of diagnostic test accuracy data. Stat Methods Med Res. 27(6):1766–1784. 27655805 10.1177/0962280216669182

[bibr18-00220345211042795] Pitts N Zero D Marsh P Ekstrand K Weintraub J Ramos-Gomez F Tagami J Twetman S Tsakos G Ismail A . 2017. Dental caries. Nat Rev Dis Primers. 3:17030. 28540937 10.1038/nrdp.2017.30

[bibr19-00220345211042795] Reitsma JB Glas AS Rutjes AW Scholten RJ Bossuyt PM Zwinderman AH . 2005. Bivariate analysis of sensitivity and specificity produces informative summary measures in diagnostic reviews. J Clin Epidemiol. 58(10):982–990. 16168343 10.1016/j.jclinepi.2005.02.022

[bibr20-00220345211042795] Roberts E Ludman AJ Dworzynski K Al-Mohammad A Cowie MR McMurray JJ Mant J ; Nice Guideline Development Group for Acute Heart Failure. 2015. The diagnostic accuracy of the natriuretic peptides in heart failure: systematic review and diagnostic meta-analysis in the acute care setting. BMJ. 350:h910. 25740799 10.1136/bmj.h910 PMC4353288

[bibr21-00220345211042795] Schünemann HJ Mustafa RA Brozek J Santesso N Bossuyt PM Steingart KR Leeflang M Lange S Trenti T Langendam M , et al. 2019. Grade guidelines: 22. The grade approach for tests and strategies-from test accuracy to patient-important outcomes and recommendations. J Clin Epidemiol. 111:69–82. 30738926 10.1016/j.jclinepi.2019.02.003

[bibr22-00220345211042795] Schünemann HJ Mustafa RA Brozek J Steingart KR Leeflang M Murad MH Bossuyt P Glasziou P Jaeschke R Lange S , et al. 2020a. Grade guidelines: 21 part 1. Study design, risk of bias, and indirectness in rating the certainty across a body of evidence for test accuracy. J Clin Epidemiol. 122:129–141. 32060007 10.1016/j.jclinepi.2019.12.020

[bibr23-00220345211042795] Schünemann HJ Mustafa RA Brozek J Steingart KR Leeflang M Murad MH Bossuyt P Glasziou P Jaeschke R Lange S , et al. 2020b. Grade guidelines: 21 part 2. Test accuracy: inconsistency, imprecision, publication bias, and other domains for rating the certainty of evidence and presenting it in evidence profiles and summary of findings tables. J Clin Epidemiol. 122:142–152. 32058069 10.1016/j.jclinepi.2019.12.021

[bibr24-00220345211042795] Schwendicke F Tzschoppe M Paris S . 2015. Radiographic caries detection: a systematic review and meta-analysis. J Dent. 43(8):924–933. 25724114 10.1016/j.jdent.2015.02.009

[bibr25-00220345211042795] Steele J O’Sullivan I . 2011. Executive summary: adult dental health survey 2009 [accessed 2021 May 8]. http://docshare01.docshare.tips/files/14940/149402877.pdf.

[bibr26-00220345211042795] Takwoingi Y Leeflang MM Deeks JJ . 2013. Empirical evidence of the importance of comparative studies of diagnostic test accuracy. Ann Intern Med. 158(7):544–554. 23546566 10.7326/0003-4819-158-7-201304020-00006

[bibr27-00220345211042795] Urquhart O Tampi MP Pilcher L Slayton RL Araujo MWB Fontana M Guzman-Armstrong S Nascimento MM Novy BB Tinanoff N , et al. 2019. Nonrestorative treatments for caries: systematic review and network meta-analysis. J Dent Res. 98(1):14–26. 30290130 10.1177/0022034518800014 PMC6304695

[bibr28-00220345211042795] Vehtari A Gelman A Gabry J . 2017. Practical Bayesian model evaluation using leave-one-out cross-validation and WAIC. Stat Comput. 27(5):1413–1432.

[bibr29-00220345211042795] Walsh T Macey R Riley P Glenny AM Schwendicke F Worthington HV Clarkson JE Ricketts D Su TL Sengupta A . 2021. Imaging modalities to inform the detection and diagnosis of early caries. Cochrane Database Syst Rev. 3:CD014545. 10.1002/14651858.CD014545 PMC8441255 33720395

[bibr30-00220345211042795] Whiting PF Rutjes AW Westwood ME Mallett S Deeks JJ Reitsma JB Leeflang MM Sterne JA Bossuyt PM ; QUADAS-2 Group. 2011. QUADAS-2: a revised tool for the quality assessment of diagnostic accuracy studies. Ann Intern Med. 155(8):529–536. 22007046 10.7326/0003-4819-155-8-201110180-00009

[bibr31-00220345211042795] Young DA Novy BB Zeller GG Hale R Hart TC Truelove EL ; American Dental Association Council on Scientific Affairs. 2015. The American Dental Association caries classification system for clinical practice: a report of the American Dental Association Council on Scientific Affairs. J Am Dent Assoc. 146(2):79–86. 25637205 10.1016/j.adaj.2014.11.018

