# Supplementary material for: Enamel Caries Detection and Diagnosis: An Analysis of Systematic Reviews
Source: J Dent Res. 2021 Oct 12;101(3):261–9. doi: 10.1177/00220345211042795 (PMC8864327; doi:10.1177/00220345211042795)
Supplement: sj-pdf-1-jdr-10.1177_00220345211042795 – Supplemental material for Enamel Caries Detection and Diagnosis: An Analysis of Systematic Reviews [file sj-pdf-1-jdr-10.1177_00220345211042795.pdf]

Enamel caries detection and diagnosis: an analysis of systematic reviews - Appendix

Tanya Walsh[1*], Richard Macey[1], David Ricketts[2], Alonso Carrasco Labra[3, 4], Helen Worthington[1], Alex J Sutton[5], Suzanne Freeman[5] , Anne-Marie Glenny[1], Phillip Riley[1], Janet Clarkson[1, 2], Enzo Cerullo[5]

1. Division of Dentistry, School of Medical Sciences, The University of Manchester, Manchester M13 9PL
2. Dundee Dental School, University of Dundee, Park Place, Dundee, DD1 4HR
3. Department of Evidence Synthesis & Translation Research, American Dental Association, Science and Research Institute, LLC, Chicago, IL, 60611
4. Department of Oral and Craniofacial Health Science, School of Dentistry, University of North Carolina at Chapel Hill, Chapel Hill, NC, 27599
5. Department of Health Sciences, University of Leicester, Lancaster Road, Leicester, UK

*** Corresponding author:**

Tanya Walsh

Division of Dentistry, Coupland 3 Building, Manchester M13 9PL, United Kingdom. E-mail tanya.walsh@manchester.ac.uk

Choice of prior distributions

For both direct comparisons and NMA models, for the mean sensitivity and specificity parameters on the logistic scale, we used normally distributed priors with mean 0 and standard deviation of 2, which corresponds to a flat prior on the probability scale. For between-study standard deviation parameters, we used half-normal priors with mean 0 and standard deviation of 1. For the between-study correlation between sensitivities and specificities, we used Lewandowki-Kurowicka-Joe (LKJ) (Lewandowski et al. 2009) priors with a shape parameter of 2. The NMA model also has between-test standard deviation parameters, where we used half-normal priors with mean 0 and standard deviation of 1. These priors allow a large variation in study-specific sensitivity and specificity if the data demands.

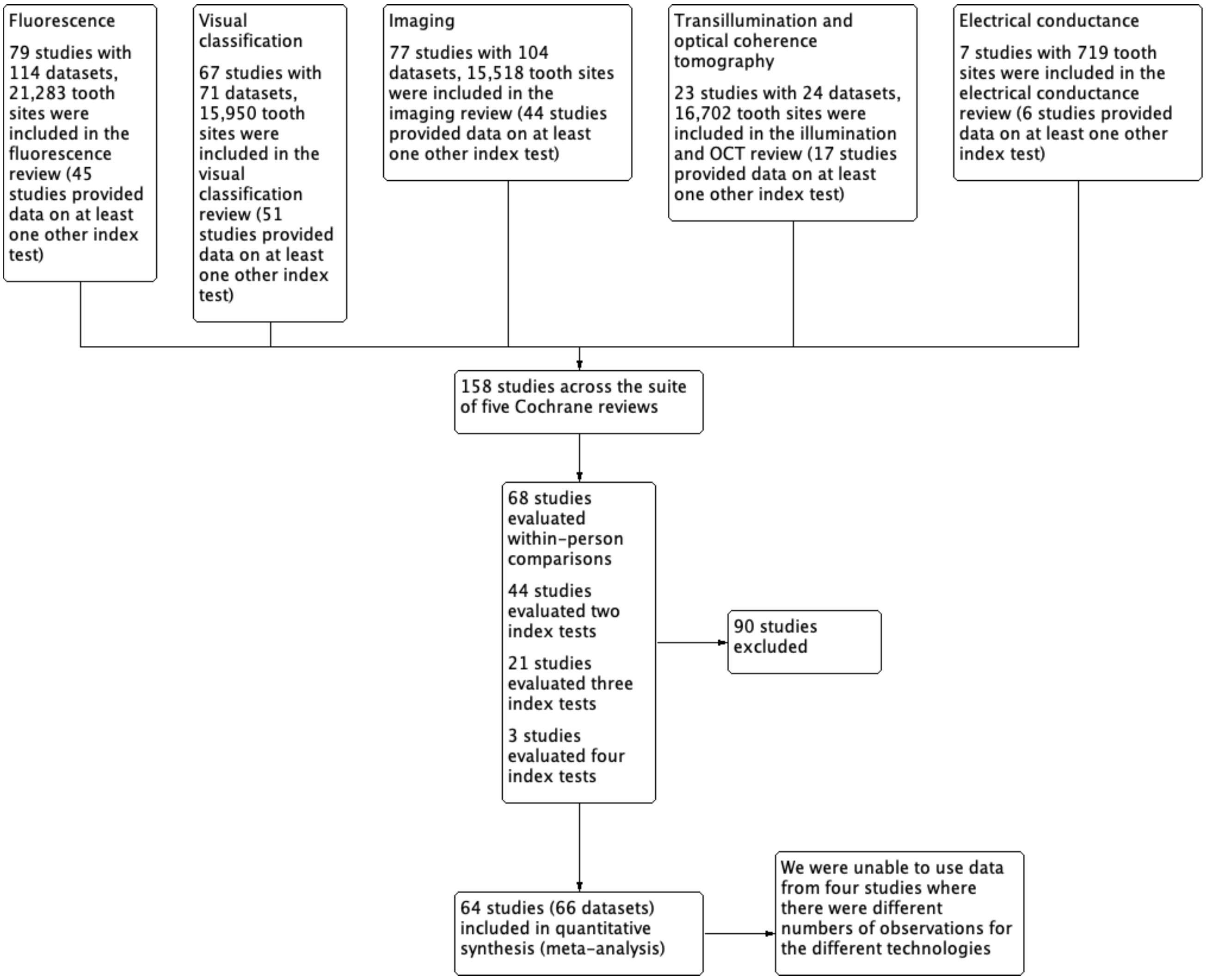

Appendix Figure 1: Flow chart of study selection NB Where a primary study provided more than one dataset per technology (e.g., analog and digital radiographs) to minimise dependency of data within an analysis the dataset with the largest volume of data was selected for inclusion. This decision was justified on the basis that for each individual systematic review no differences in accuracy estimates were typically observed within the individual technologies.

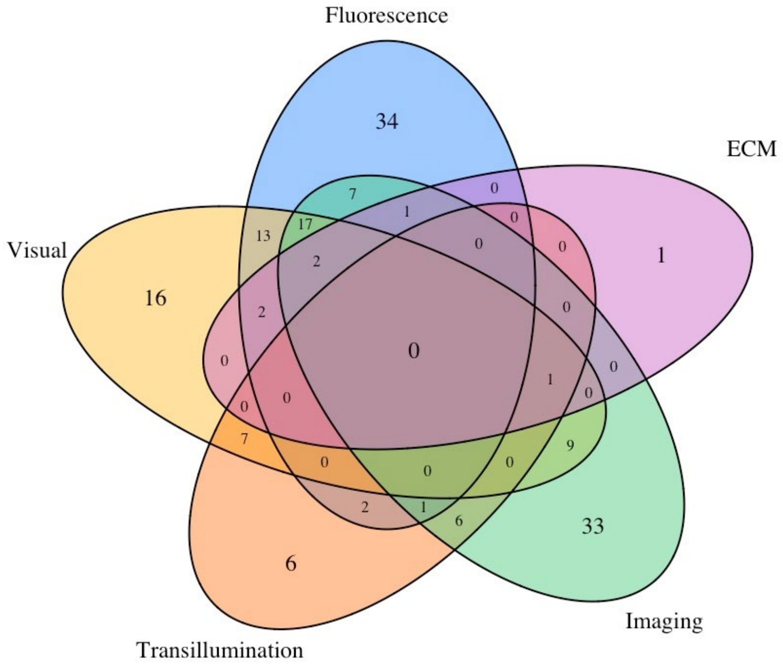

Appendix Figure 2: Distribution of how many studies compared each combination of specific technologies across the Cochrane systematic reviews (N.B. All studies reporting one technology only (indicated in each ellipse towards the edge of the figure) were excluded from this review.

Study ID	Technology	Tooth sites	Teeth	Participants	In-vitro In-vivo	Prevalence D_1	Prevalence D_3	Surface	Ref standard	Dentition
1. (Achilleos et al. 2013)	Fluorescence (red fluorescence)<br>Visual (ICDAS)	38	38	NR - extracted	vitro	0.95	0.39	Occlusal	Histology	Permanent
2. (Akarsu and Koprulu 2006)	Fluorescence (red fluorescence)<br>Visual (Ekstrand/ERK)	165	187	161	vivo	0.77	0.52	Occlusal	Excavation	Permanent
3. (Apostolopoulou et al. 2009)	Fluorescence (red fluorescence)<br>Imaging (analog radiograph)	111	24	NR - extracted	vitro	0.98	0.22	Occlusal	Histology	Primary
4. (Ashley et al. 1998)	Visual (other)<br>Imaging (analog radiograph)<br>Electrical conductance / impedance<br>Transillumination (FOTI/DIFOTI)	103	103	NR - extracted	vitro	0.60	0.36	Occlusal	Histology	Permanent
5. (Astvaldsdottir et al. 2012)	Imaging (analog radiograph)<br>Transillumination (FOTI/DIFOTI)	56	112	NR - extracted	vitro	0.64	0.15	Approximal	Histology	Permanent
6. (Attrill and Ashley 2001)	Fluorescence (red fluorescence)<br>Imaging (analog radiograph)	58	58	NR - extracted	vitro	0.60	0.51	Occlusal	Histology	Primary
7. (Bahrololoomi et al. 2015)	Fluorescence (red fluorescence)<br>Visual (Ekstrand/ERK)<br>Imaging (analog radiograph)	109	115	31	vivo	0.94	0.52	Occlusal	Excavation	Permanent
8. (Braga et al. 2009)	Fluorescence (red fluorescence)<br>Visual (ICDAS)<br>Imaging (analog radiograph)	131	84	NR - extracted	vitro	0.63	0.26	Occlusal	Histology	Primary
9. (Braun et al. 2017)	Visual (ICDAS)<br>Imaging (digital radiograph)	84	84	NR - extracted	vitro	0.77	0.40	Occlusal	Histology	Primary
10. (Bussaneli et al. 2015c)	Transillumination (NIR)<br>Fluorescence (red fluorescence)<br>Imaging (analog radiograph)	94	102	NR - extracted	vitro	0.70	0.19	Occlusal	Histology	Permanent
11. (Bussaneli et al. 2015b)	Fluorescence (red fluorescence)<br>Visual (other)	59	59	45	vitro	0.71	0.58	Approximal	Visual	Primary

Study ID	Technology	Tooth sites	Teeth	Participants	In-vitro In-vivo	Prevalence D_1	Prevalence D_3	Surface	Ref standard	Dentition
12. (Bussaneli et al. 2015a)	Visual (ICDAS)<br>Imaging (analog radiograph)	77	77	NR - extracted	vitro	0.63	NR	Occlusal	Histology	Permanent
13. (Castilho et al. 2016)	Fluorescence (red fluorescence)<br>Visual (ICDAS)	43	43	26	vivo	0.81	0.07	Occlusal	Histology	Permanent
14. (Chawla et al. 2012)	Visual (ICDAS)<br>Transillumination (FOTI/DIFOTI)	135	NR	NR - extracted	vitro	0.61	0.24	Approximal	Histology	Primary
15. (Cinar et al. 2013)	Fluorescence (red fluorescence)<br>Visual (Ekstrand/ERK)<br>Imaging (analog radiograph)	44	NR	NR - extracted	vitro	0.75	0.2	Occlusal	Histology	Primary
16. (Costa et al. 2002)	Fluorescence (red fluorescence)<br>Visual (other)<br>Imaging (digital radiograph)	49	49	NR - extracted	vitro	0.65	0.31	Occlusal	Histology	Permanent
17. (Dias da Silva et al. 2010)	Visual (Ekstrand/ERK)<br>Imaging (digital radiograph)	50	50	NR - extracted	Vitro	0.56	0.38	Occlusal	Histology	Primary
18. (Diniz et al. 2011)	Visual (ICDAS)<br>Imaging (analog radiograph)	104	104	NR - extracted	vitro	0.94	NR	Occlusal	Histology	Permanent
19. (Diniz et al. 2012)	Fluorescence (red fluorescence)<br>Visual (ICDAS)<br>Imaging (analog radiograph)	105	105	88	vitro	0.95	0.26	Occlusal	Histology	Permanent
20. (Diniz et al. 2019)	Fluorescence (red fluorescence)<br>Visual (ICDAS)	88	88	NR - extracted	vitro	0.75	0.63	Occlusal	Histology	Primary
21. (Ekstrand et al. 2011)	Visual (ICDAS)<br>Imaging (analog radiograph)	160	140	NR - extracted	vitro	0.82	0.64	Approximal	Histology	Permanent
22. (Freitas et al. 2016)	Visual (ICDAS)<br>Imaging (analog radiograph)	166	89	56	vivo	0.68	0.5	Approximal	Histology	Primary
23. (Goel et al. 2009)	Fluorescence (red fluorescence)<br>Visual (Ekstrand/ERK)<br>Imaging (analog radiograph)	83	84	NR	vivo	0.98	0.43	Occlusal	Histology	Permanent
24. (Hintze and Wenzel 2003)	Visual (other)<br>Imaging (analog radiograph)	373	198	NR - extracted	vitro	0.56	0.14	Approximal	Histology	Permanent

Study ID	Technology	Tooth sites	Teeth	Participants	In-vitro In-vivo	Prevalence D_1	Prevalence D_3	Surface	Ref standard	Dentition
25. (Huth et al. 2010)	Fluorescence (red fluorescence)<br>Visual (Ekstrand/ERK)	117	117	117	vivo	0.66	0.37	Occlusal	Excavation	Permanent
26. (Iranzo-Cortes et al. 2017)	Fluorescence (red fluorescence)<br>Visual (ICDAS)	64	65	NR - extracted	vitro	0.77	0.17	Occlusal	Histology	Permanent
27. (Jablonski-Momeni et al. 2012)	Fluorescence (blue fluorescence)<br>Visual (ICDAS)	80	36	NR - extracted	vitro	0.84	0.48	Occlusal	Histology	Permanent
28. (Jablonski-Momeni et al. 2017)	Imaging (digital radiograph)<br>Transillumination (NIR)	193	161	18	vivo	0.62	NR	Approximal	Visual	Permanent
29. (Fabian et al.)	Imaging (digital radiograph)<br>Transillumination (NIR)	70	70	35	vivo	0.56	0.03	Approximal	Visual	Permanent
30. (Kim et al. 2017)	Fluorescence (green fluorescence)<br>Visual (other)	280	280	65	vitro	0.61	0.2	Approximal	Radiograph	Permanent
31. (Ko et al. 2015)	Fluorescence (green fluorescence)<br>Visual (ICDAS)<br>Imaging (digital radiograph)	95	120	NR	vivo	0.80	0.15	Approximal	Histology	Permanent
32. (Kockanat and Unal 2017)	Fluorescence (red fluorescence)<br>Visual (ICDAS)<br>Imaging (digital radiograph)<br>Electrical conductance / impedance	120	144	NR	vivo	0.78	0.32	Occlusal	Histology	Primary
33. (Kucukyilmaz et al. 2015)	Fluorescence (red fluorescence)<br>Visual (other)<br>Imaging (digital radiograph)<br>Electrical conductance / impedance	200	200	200	vivo	0.82	0.33	Occlusal	Histology	Primary
34. (Laitala et al. 2017)	Visual (ICDAS)<br>Transillumination (FOTI/DIFOTI)	2103	1162	91	vivo	0.20	0.06	Approximal	Radiograph	Permanent

Study ID	Technology	Tooth sites	Teeth	Participants	In-vitro In-vivo	Prevalence D_1	Prevalence D_3	Surface	Ref standard	Dentition
35. (Lussi et al. 2006)	Fluorescence (red fluorescence)<br>Imaging (analog radiograph)	150	150	75	vitro	0.59	0.25	Approximal	Histology	Permanent
36. (Mansour et al. 2016)	Fluorescence (red fluorescence)<br>OCT	426	932	40	vitro	0.12	0.14	Occlusal	Visual	Permanent
37. (Matos et al. 2011)	Fluorescence (red fluorescence)<br>Imaging (analog radiograph)	382	382	68	vivo	0.92	0.05	Occlusal	Visual	Primary
38. (Mendes et al. 2006)	Fluorescence (red fluorescence)<br>Visual (Ekstrand/ERK)<br>Imaging (analog radiograph)	110	79	NR - extracted	vitro	0.75	0.25	Occlusal	Histology	Primary
39. (Mialhe et al. 2003)	Imaging (analog radiograph)<br>Transillumination (FOTI/DIFOTI)	199	199	70	vivo	0.85	0.14	Approximal	Visual	Permanent
40. (Mitropoulos et al. 2010)	Visual (ICDAS)<br>Imaging (analog radiograph)	410	20	NR - extracted	vitro	0.60	0.45	Approximal	Histology	Permanent
41. (Mortensen et al. 2018)	Fluorescence (red fluorescence)<br>Imaging (digital radiograph)<br>Electrical conductance / impedance	60	57	57	vivo	0.97	0.45	Occlusal	Visual	Permanent
42. (Nakagawa et al. 2013)	Visual (other)<br>OCT	127	93	NR - extracted	vitro	0.75	0.31	Smooth	Visual	NR
43. (Nakajima et al. 2014)	Visual (other)<br>OCT	38	26	NR - extracted	vitro	0.74	0.32	Occlusal	Histology	Primary
44. (Neuhaus et al. 2011)	Fluorescence (red fluorescence)<br>Visual (ICDAS)<br>Imaging (analog radiograph)	37	37	NR - extracted	vitro	0.73	0.24	Occlusal	Histology	Primary
45. (Novaes et al. 2009)	Fluorescence (red fluorescence)<br>Visual (ICDAS)<br>Imaging (analog radiograph)	621	50	NR	vivo	0.41	0.03	Approximal	Visual	Primary
46. (Novaes et al. 2010)	Fluorescence (red fluorescence)<br>Visual (ICDAS)<br>Imaging (analog radiograph)	592	168	76	vivo	0.81	0.05	Approximal	Visual	Primary

Study ID	Technology	Tooth sites	Teeth	Participants	In-vitro In-vivo	Prevalence D_1	Prevalence D_3	Surface	Ref standard	Dentition
47. (Novaes et al. 2012)	Visual (ICDAS)<br>Imaging (analog radiograph)	344	76	76	vivo	0.80	NR	Approximal	Visual	Primary
48. (de Paula et al. 2011)	Fluorescence (red fluorescence)<br>Visual (other)	64	64	26	vitro	0.88	0.28	Occlusal	Histology	Permanent
49. (Pereira Antônio et al. 2011)	Fluorescence (red fluorescence)<br>Visual (Ekstrand/ERK)<br>Electrical conductance / impedance	96	96	NR - extracted	vitro	0.57	0.25	Occlusal	Histology	Permanent
50. (Rocha et al. 2003)	Fluorescence (red fluorescence)<br>Visual (Ekstrand/ERK)<br>Imaging (analog radiograph)	100	50	29	vivo	0.58	0.14	Occlusal	Histology	Primary
51. (Rodrigues et al. 2008)	Fluorescence (blue fluorescence)<br>Visual (ICDAS)<br>Imaging (analog radiograph)	119	119	NR - extracted	vitro	0.93	0.54	Occlusal	Histology	Permanent
52. (Rodrigues et al. 2009)	Fluorescence (red fluorescence)<br>Visual (other)	148	148	NR - extracted	vitro	0.92	0.03	Occlusal	Histology	Primary
53. (Seremidi et al. 2012)	Fluorescence (red fluorescence)<br>Visual (Ekstrand/ERK)	107	107	41	vitro	0.78	0.19	Occlusal	Histology	Permanent
54. (Shi et al. 2000)	Fluorescence (red fluorescence)<br>Visual (other)	70	76	NR - extracted	vitro	0.73	0.39	Occlusal	Histology	Permanent
55. (Shimada et al. 2010)	Visual (Ekstrand/ERK)<br>OCT	111	62	NR - extracted	vitro	0.86	0.38	Occlusal	Histology	Permanent
56. (Shimada et al. 2014)	Imaging (analog radiograph)<br>OCT	91	53	53	vivo	0.67	0.38	Approximal	Excavation	Permanent
57. (Sidi and Naylor 1988)	Visual (other)<br>FOTI	4405	4405	456	vivo	0.03	0.01	Approximal	Radiographs	Permanent
58. (Simon et al. 2016)	Imaging (digital radiograph)<br>Transillumination (NIR)	109	40	40	vivo	0.82	NR	Occlusal	Histology	Permanent

Study ID	Technology	Tooth sites	Teeth	Participants	In-vitro In-vivo	Prevalence D_1	Prevalence D_3	Surface	Ref standard	Dentition
59. (Souza et al. 2013)	Fluorescence (red fluorescence)<br>Visual (ICDAS)<br>Imaging (analog radiograph)	79	79	NR - extracted	vitro	0.76	0.35	Occlusal	Histology	Permanent
60. (de Souza et al. 2014)	Fluorescence (red fluorescence)<br>Imaging (analog radiograph)	102	51	NR - extracted	vitro	0.48	0.34	Approximal	Histology	Permanent
61. (de Souza et al. 2018)	Fluorescence (red fluorescence)<br>Imaging (digital radiograph)	195	195	46	vitro	0.41	0.13	Approximal	Visual	Primary
62. (Soviero et al. 2012)	Visual (ICDAS)<br>Imaging (analog radiograph)	48	25	NR - extracted	vitro	0.81	0.33	Approximal	Histology	Primary
63. (Sridhar et al. 2009)	Fluorescence (red fluorescence)<br>Visual (Ekstrand/ERK)	50	50	NR - extracted	vitro	0.88	0.12	Occlusal	Histology	Permanent
64. (Teo et al. 2014)	Fluorescence (red fluorescence)<br>Visual (ICDAS)<br>Electrical conductance / impedance	64	64	NR - extracted	vitro	0.72	0.31	Occlusal	Histology	Permanent
65. (Tonkaboni et al. 2018)	Fluorescence (blue fluorescence)<br>Visual (ICDAS)<br>Imaging (analog radiograph)	108	108	NR - extracted	vitro	0.43	0.35	Approximal	Histology	Permanent
66. (Van Hilsen and Jones 2013)	Fluorescence (red fluorescence)<br>OCT	42	45	NR - extracted	vitro	0.76	0.31	Occlusal	Histology	Permanent
67. (Virajsilp et al. 2005)	Fluorescence (red fluorescence)<br>Imaging (analog radiograph)	107	72	NR - extracted	vitro	0.83	0.5	Approximal	Histology	Primary
68. (Xiao-Hua et al. 2016)	Visual (Ekstrand/ERK)<br>OCT	97	77	NR - extracted	vitro	0.74	0.48	Occlusal	Histology	Permanent

NR Not reported

Appendix Table 1: Characteristics of included studies

Assessment of risk of bias for the reference standard.

The lack of a ‘perfect’ reference standard for caries detection and diagnosis is a particular challenge when designing and conducting studies of diagnostic test accuracy in this area, and imperfect reference standards are therefore employed. The

Cochrane reviews considered histology, radiographs, excavation, or enhanced visual examination (+/- tooth separation) as eligible reference standards. To ensure comparability and minimise bias, each tooth site or surface within a study would be exposed to the same reference test. This is easier to achieve with in vitro studies where, for example, a reference standard of histology can be applied to each tooth. In vivo studies may have applied the same reference standard by using enhanced visual examination or radiographs as a reference standard. Where a study allocated participants or tooth sites / surfaces to different reference standards this should be clearly reported and justified.

Ideally, the index test and reference standard would have been undertaken by different, blinded examiners, and all reference standards should have been completed without knowledge of the index test results. Where studies used extracted teeth that had been sectioned and prepared for histological evaluation it is unlikely that the same examiner would be able to recall results from the index test for the specific tooth under evaluation.

	Risk of bias assessment								Applicability assessment						
Study ID	Patient selection	Index test fluorescence	Index test trans-illumination & OCT	Index test visual classification	Index test imaging	Index test electrical conductance	Reference standard	Flow & timing	Patient selection	Index test fluorescence	Index test trans-illumination & OCT	Index test visual classification	Index test imaging	Index test electrical conductance	Reference standard
1. (Achilleos et al. 2013)	H	L		L			L	L	H	L		H			L
2. (Akarsu and Koprulu 2006)	U	H		L			H	H	L	L		L			L
3. (Apostolopoulou et al. 2009)	H	U			L		L	L	H	L			U		L
4. (Ashley et al. 1998)	H		L	L	U	U	L	L	H		L	H	L	L	L
5. (Astvaldsdottir et al. 2012)	H		U		L		L	L	H		L		L		L
6. (Attrill and Ashley 2001)	H	L			L		L	L	H	L			H		L
7. (Bahrololoomi et al. 2015)	H	H		H	H		U	H	L	U		L	U		L
8. (Braga et al. 2009)	H	H		U	H		L	L	H	L		H	L		L
9. (Braun et al. 2017)	U			L	H		L	L	H			L	H		L
10. (Bussaneli et al. 2015c)	H	L	U		H		L	H	H	L	H		H		L
11. (Bussaneli et al. 2015b)	H	L		L	L		H	L	H	L		L	L		H
12. (Bussaneli et al. 2015a)	H			L	H		U	L	H			H	L		L
13. (Castilho et al. 2016)	L	L		L			L	L	L	L		L			L
14. (Chawla et al. 2012)	H	L	L	L	L		L	L	H	L	L	H	L		L
15. (Cinar et al. 2013)	H	L		L	H		L	L	L	L		L	L		L
16. (Costa et al. 2002)	H	L		L	H		L	L	H	L		H	L		L
17. (Dias da Silva et al. 2010)	H			L	H		L	L	H			H	H		L
18. (Diniz et al. 2011)	H			L	L		L	L	H			H	H		L
19. (Diniz et al. 2012)	U	H		L	H		L	L	H	L		L	L		L
20. (Diniz M et al. 2019)	U	H		L			U	L	H	L		H			L
21. (Ekstrand et al. 2011)	H			L	L		L	L	H			H	U		H
22. (Freitas et al. 2016)	H			L	H		L	U	L			L	L		L
23. (Goel et al. 2009)	H	L		U	U		L	L	L	L		L	U		L
24. (Hintze and Wenzel 2003)	H			L	U		L	L	H			H	L		L
25. (Huth et al. 2010)	L	H		L			H	H	L	L		L			L
26. (Iranzo-Cortes et al. 2017)	H	L		L			L	L	H	L		H			L
27. (Jablonski-Momeni et al. 2012)	U	U		L	U		L	L	H	L		H	H		L
28. (Jablonski-Momeni et al. 2017)	U		L	U	U		H	L	L		L	L	L		L
29. (Fabian et al.)	H		L		H		H	L	L		L		L		L
30. (Kim et al. 2017)	U	H		L			H	H	H	L		L			L
31. (Ko et al. 2015)	H	H		L	H		L	L	H	L		L	L		L

32. (Kockanat and Unal 2017)	U	L		L	L	L	L	H	L	L		H	L	L	L
33. (Kucukyilmaz et al. 2015)	H	L		L	H	L	L	L	L	L		L	L	L	L
34. (Laitala et al. 2017)	U		U	L	L		H	L	L		L	L	L		L
35. (Lussi et al. 2006)	H	H			L		L	L	H	L			H		L
36. (Mansour et al. 2016)	U	U	U				H	L	L	U	U				L
37. (Matos et al. 2011)	L	H			H		H	H	L	L			L		L
38. (Mendes et al. 2006)	H	H		U	L		L	L	H	L		H	H		L
39. (Mialhe et al. 2003)	U		L		L		H	H	L		L		L		L
40. (Mitropoulos et al. 2010)	H			L	L		L	L	H			H	H		L
41. (Mortensen et al. 2018)	H	L			H	U	H	L	U	L			L	L	L
42. (Nakagawa et al. 2013)	H		L	L			L	L	L		L	H			L
43. (Nakajima et al. 2014)	H		L	L			L	L	H		L	H			L
44. (Neuhaus et al. 2011)	H	H		H	L		U	L	H	L		H	H		L
45. (Novaes et al. 2009)	L	H		L	H		H	L	L	L		L	L		L
46. (Novaes et al. 2010)	L	L		L	H		H	L	L	L		L	L		L
47. (Novaes et al. 2012)	L	U		U	U		H	L	H	L		H	L		L
48. (de Paula et al. 2011)	H	L		L			L	L	H	L		H			L
49. (Pereira Antônio et al. 2011)	H	L		L		L	L	L	H	L		H		L	L
50. (Rocha et al. 2003)	H	L		H	H		L	L	H	L		L	L		L
51. (Rodrigues et al. 2008)	H	L		L	H		L	L	H	L		H	L		L
52. (Rodrigues et al. 2009)	H	L		L			L	L	H	L		H			L
53. (Seremidi et al. 2012)	H	L		L			L	L	H	L		H			L
54. (Shi et al. 2000)	H	H		H	H		L	H	H	L		H	L		L
55. (Shimada et al. 2010)	H		L	L			L	L	H		L	H			L
56. (Shimada et al. 2014)	H		L	L	L		H	H	L		L	L	L		L
57. (Sidi and Naylor 1988)	H		U	U	U		H	U	H		L	U	L		H
58. (Simon et al. 2016)	U		L		L		L	L	L		H		L		L
59. (Souza J et al. 2013)	H	L		L	H		L	L	L	L		H	L		L
60. (de Souza et al. 2014)	H	H			L		L	L	H	L			H		L
61. (de Souza et al. 2018)	L	L			L		H	L	H	L			L		L
62. (Soviero et al. 2012)	H			L	H		L	L	H			H	L		L
63. (Sridhar et al. 2009)	H	L		L			L	L	L	L		H			L
64. (Teo et al. 2014)	U	L		L		L	L	L	L	L		L		L	L
65. (Tonkaboni et al. 2018)	U	U		L	U		U	L	H	L		L	L		L
66. (Van Hilsen and Jones 2013)	H	L	L				L	L	H	L	L				L
67. (Virajsilp et al. 2005)	H	H			H		L	L	H	L			L		L
68. (Xiao-Hua et al. 2016)	H		L	L			L	L	H		L	H			L

Appendix Table 2: QUADAS-2 assessments from the original Cochrane reviews.

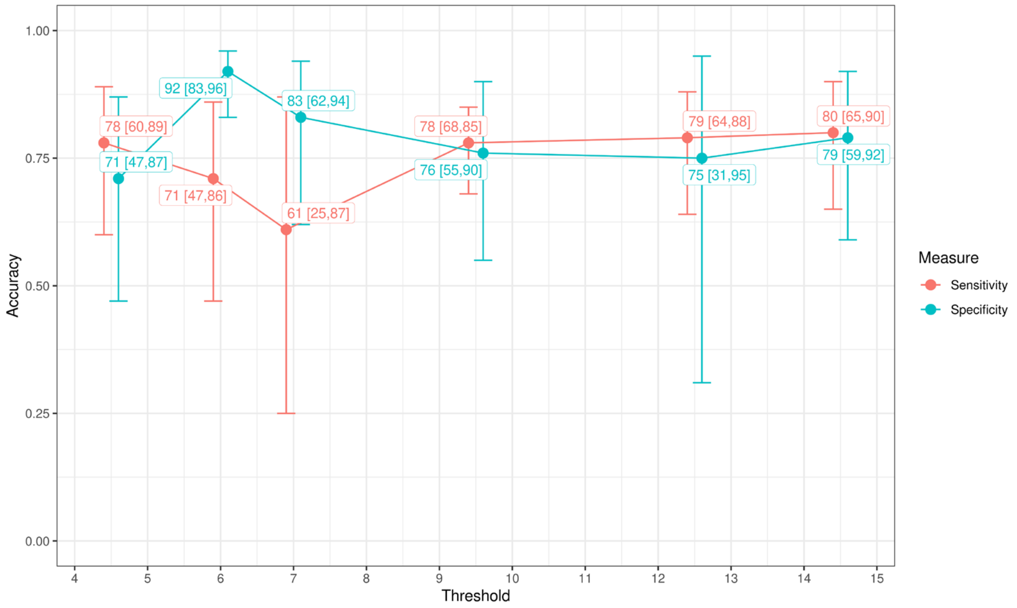

Appendix Figure 3: Stratified bivariate analysis for fluorescence-based technologies reporting on a continuous scale and grouping studies with similar thresholds.

Appendix References

Achilleos EE, Rahiotis C, Kakaboura A, Vougiouklakis G. 2013. Evaluation of a new fluorescence-based device in the detection of incipient occlusal caries lesions. Lasers in medical science. 28(1):193-201.

Akarsu S, Koprulu H. 2006. In vivo comparison of the efficacy of diagnodent by visual inspection and radiographic diagnostic techniques in the diagnosis of occlusal caries. Journal of clinical dentistry. 17(3):53-58.

Apostolopoulou D, Lagouvardos P, Kavvadia K, Papagiannoulis L. 2009. Histological validation of a laser fluorescence device for occlusal caries detection in primary molars. European archives of paediatric dentistry: official journal of the european academy of paediatric dentistry. 10 Suppl 1:11-15.

Ashley PF, Blinkhorn AS, Davies RM. 1998. Occlusal caries diagnosis: An in vitro histological validation of the electronic caries monitor (ecm) and other methods. Journal of dentistry. 26(2):83-88.

Astvaldsdottir A, Ahlund K, Holbrook W P, de Verdier B, Tranaeus S. 2012. Approximal caries detection by difoti: In vitro comparison of diagnostic accuracy/efficacy with film and digital radiography. International journal of dentistry. 2012:326401-326401.

Attrill DC, Ashley PF. 2001. Occlusal caries detection in primary teeth: A comparison of diagnodent with conventional methods. British dental journal. 190(8):440-443.

Bahrololoomi Z, Ezoddini F, Halvani N. 2015. Comparison of radiography, laser fluorescence and visual examination for diagnosing incipient occlusal caries of permanent first molars. Journal of dentistry / tehran university of medical sciences. 12(5):324-332.

Braga M, Morais C, Nakama R, Leamari V, Siqueira W, Mendes F. 2009. In vitro performance of methods of approximal caries detection in primary molars. Oral surgery, oral medicine, oral pathology, oral radiology and endodontology. 108(4):e35-e41.

Braun A, Guiraud LM, Frankenberger R. 2017. Histological validation of icdas ii and radiological assessment of occlusal carious lesions in permanent teeth. Odontology. p. 46-53.

Bussaneli DG, Boldieri T, Diniz M B, Rivera L M, Santos-Pinto L, Cordeiro Rde C. 2015a. Influence of professional experience on detection and treatment decision of occlusal caries lesions in primary teeth. International journal of paediatric dentistry. 25(6):418-427.

Bussaneli DG, Restrepo M, Boldieri T, Albertoni T H, Santos-Pinto L, Cordeiro R C L. 2015b. Proximal caries lesion detection in primary teeth: Does this justify the association of diagnostic methods? Lasers in medical science. 30(9):2239-2244.

Bussaneli DG, Restrepo M, Boldieri T, Pretel H, Mancini MW, Santos-Pinto L, et a. 2015c. Assessment of a new infrared laser transillumination technology (808 nm) for the detection of occlusal caries-an in vitro study. Lasers in medical science. 30(7):1873-1879.

Castilho LS, Cotta F V, Bueno A C, Moreira A N, Ferreira E F, Magalhaes CS. 2016. Validation of diagnodent laser fluorescence and the international caries detection and assessment system (icdas) in diagnosis of occlusal caries in permanent teeth: An in vivo study. European journal of oral sciences. 124(2):188-194.

Chawla N, Messer LB, Adams GG, Manton DJ. 2012. An in vitro comparison of detection methods for approximal carious lesions in primary molars. . Caries Research 46(2):161-169.

Cinar C, Atabek D, Odabas M E, Olmez A. 2013. Comparison of laser fluorescence devices for detection of caries in primary teeth. International dental journal. 63(2):97-102.

Costa AM, Yamaguti PM, De Paula LM, Bezerra AC. 2002. In vitro study of laser diode 655 nm diagnosis of occlusal caries. Journal of dentistry for children. 69(3):249-253, 233.

de Paula AB, Campos JA, Diniz MB, Hebling J, Rodrigues JA. 2011. In situ and in vitro comparison of laser fluorescence with visual inspection in detecting occlusal caries lesions. Lasers Med Sci. 26(1):1-5.

de Souza JF, Diniz MB, Boldieri T, Rodrigues JA, Lussi A, de Cassia Loiola Cordeiro R. 2014. In vitro performance of a pen-type laser fluorescence device and bitewing radiographs for approximal caries detection in permanent and primary teeth. Indian J Dent Res. 25(6):702-710.

de Souza L, Cancio V, Tostes M. 2018. Accuracy of pen-type laser fluorescence device and radiographic methods in detecting approximal carious lesions in primary teeth - an in vivo study. International journal of paediatric dentistry / the British Paedodontic Society [and] the International Association of Dentistry for Children.

Dias da Silva PR, Martins Marques M, Steagall W, Jr., Medeiros Mendes F, Lascala CA. 2010. Accuracy of direct digital radiography for detecting occlusal caries in primary teeth compared with conventional radiography and visual inspection: An in vitro study. Dentomaxillofac Radiol. 39(6):362-367.

Diniz M, Campos P, Wilde S, Cordeiro R, Zandona A. 2019. Performance of light-emitting diode device in detecting occlusal caries in the primary molars. Lasers Med Sci. 34(6):1235-1241.

Diniz MB, Boldieri T, Rodrigues JA, Santos-Pinto L, Lussi A, Cordeiro RC. 2012. The performance of conventional and fluorescence-based methods for occlusal caries detection: An in vivo study with histologic validation. . Journal of the American Dental Association 143(4):339-350.

Diniz MB, Lima LM, Eckert G, Zandona AG, Cordeiro RC, Pinto LS. 2011. In vitro evaluation of icdas and radiographic examination of occlusal surfaces and their association with treatment decisions. Operative dentistry. 36(2):133-142.

Ekstrand KR, Luna LE, Promisiero L, Cortes A, Cuevas S, Reyes J F, et a. 2011. The reliability and accuracy of two methods for proximal caries detection and depth on directly visible proximal surfaces: An in vitro study. Caries research. 45(2):93-99.

Fabian NJ, Adrian L, Klaus WN. Validation of near-infrared transillumination for early approximal caries detection: A prospective clinical trial.

Freitas LA, Santos MT, Guare RO, et a. 2016. Association between visual inspection, caries activity status, and radiography with treatment decisions on approximal caries in primary molars. European journal of paediatric dentistry. p. 99-104.

Goel A, Chawla H S, Gauba K, Goyal A. 2009. Comparison of validity of diagnodent with conventional methods for detection of occlusal caries in primary molars using the histological gold standard: An in vivo study. Journal of the Indian Society of Pedodontics and Preventive Dentistry. 27(4):227-234.

Hintze H, Wenzel A. 2003. Diagnostic outcome of methods frequently used for caries validation. A comparison of clinical examination, radiography and histology following hemisectioning and serial tooth sectioning. Caries research. 37(2):115-124.

Huth KC, Lussi A, Gygax M, Thum M, Crispin A, Paschos Eea. 2010. In vivo performance of a laser fluorescence device for the approximal detection of caries in permanent molars. . Journal of Dentistry. 38(12):1019-1026.

Iranzo-Cortes JE, Terzic S, Montiel-Company JM, Almerich-Silla JM. 2017. Diagnostic validity of icdas and diagnodent combined: An in vitro study in pre-cavitated lesions. Lasers in medical science. 32(3):543-548.

Jablonski-Momeni A, Jablonski B, Lippe N. 2017. Clinical performance of the near-infrared imaging system vistacam ix proxi for detection of approximal enamel lesions. Bdj open 2017;3:17012. BDJ Open. 3:17012.

Jablonski-Momeni A, Stucke J, Steinberg T, Heinzel-Gutenbrunner M. 2012. Use of icdas-ii, fluorescence-based methods, and radiography in detection and treatment decision of occlusal caries lesions: An in vitro study. International journal of dentistry. 2012:371595-371595.

Kim ES, Lee ES, Kang SM, Jung EH, de Josselin de Jong E, Jung HI, et al. 2017. A new screening method to detect proximal dental caries using fluorescence imaging. Photodiagnosis and photodynamic therapy. 20:257-262.

Ko H, Kang S, Kim H, Kwon H, Kim B. 2015. Validation of quantitative light-induced fluorescence-digital (qlf-d) for the detection of approximal caries in vitro. Journal of dentistry. 43(5):568-575.

Kockanat A, Unal M. 2017. In vivo and in vitro comparison of icdas ii, diagnodent pen, cariescan pro and soprolife camera for occlusal caries detection in primary molar teeth. European journal of paediatric dentistry. 18(2):99-104.

Kucukyilmaz E, Sener Y, Botsali MS. 2015. In vivo and in vitro performance of conventional methods, diagnodent, and an electronic caries monitor for occlusal caries detection in primary teeth. Pediatric dentistry. 37(4):E14-E22.

Laitala ML, Piipari L, Sampi N, Korhonen M, Pesonen P, Joensuu Tea. 2017. Validity of digital imaging of fiber-optic transillumination in caries detection on proximal tooth surfaces. International journal of dentistry. 2017:8289636-8289636.

Lewandowski D, Kurowicka D, Joe H. 2009. Generating random correlation matrices based on vines and extended onion method. Journal of Multivariate Analysis. 100(9):1989-2001.

Lussi A, Hack A, Hug I, Heckenberger H, Megert B, Stich H. 2006. Detection of approximal caries with a new laser fluorescence device. Caries research. 40(2):97-103.

Mansour S, Ajdaharian J, Nabelsi T, Chan G, Wilder-Smith P. 2016. Comparison of caries diagnostic modalities: A clinical study in 40 subjects. Lasers in surgery and medicine. 48(10):924-928.

Matos R, Novaes TF, Braga MM, Siqueira WL, Duarte DA, Mendes FM. 2011. Clinical performance of two fluorescence-based methods in detecting occlusal caries lesions in primary teeth. Caries research. 45(3):294-302.

Mendes FM, Ganzerla E, Nunes A F, Puig A V, Imparato JC. 2006. Use of high-powered magnification to detect occlusal caries in primary teeth. American journal of dentistry. 19(1):19-22.

Mialhe FL, Pereira AC, Pardi V, de Castro Meneghim M. 2003. Comparison of three methods for detection of carious lesions in proximal surfaces versus direct visual examination after tooth separation. Journal of clinical pediatric dentistry. 28(1):59-62.

Mitropoulos P, Rahiotis C, Stamatakis H, Kakaboura A. 2010. Diagnostic performance of the visual caries classification system icdas ii versus radiography and micro-computed tomography for proximal caries detection: An in vitro study. Journal of dentistry. 38(11):859-867.

Mortensen D, Hessing-Olsen I, Ekstrand K R, Twetman S. 2018. In-vivo performance of impedance spectroscopy, laser fluorescence, and bitewing radiographs for occlusal caries detection. Quintessence international. 49(4):293-299.

Nakagawa H, Sadr A, Shimada Y, Tagami J, Sumi Y. 2013. Validation of swept source optical coherence tomography (ss-oct) for the diagnosis of smooth surface caries in vitro. Journal of dentistry. 41:80-89.

Nakajima Y, Shimada Y, Sadr A, Wada I, Miyashin M, Takagi Y, et a. 2014. Detection of occlusal caries in primary teeth using swept source optical coherence tomography. Journal of biomedical optics. 19:16020-16020.

Neuhaus KW, Rodrigues JA, Hug I, Stich H, Lussi A. 2011. Performance of laser fluorescence devices, visual and radiographic examination for the detection of occlusal caries in primary molars. Clinical oral investigations. 15(5):635-641.

Novaes TF, Matos R, Braga MM, Imparato JC, Raggio DP, Mendes FM. 2009. Performance of a pen-type laser fluorescence device and conventional methods in detecting approximal caries lesions in primary teeth--in vivo study. Caries research. 43(1):36-42.

Novaes TF, Matos R, Gimenez T, Braga MM, Mendes F M. 2012. Performance of fluorescence-based and conventional methods of occlusal caries detection in primary molars - an in vitro study. International journal of paediatric dentistry. 22(6):459-466.

Novaes TF, Matos R, Raggio DP, Imparato JC, Braga MM, Mendes FM. 2010. Influence of the discomfort reported by children on the performance of approximal caries detection methods. Caries research. 44(5):465-471.

Pereira Antônio C, Eggertsson H, González-Cabezas C, Zero Domenick T, Eckert George J, Mialhe Fábio L. 2011. Quantitative light-induced fluorescence (qlf) in relation to other technologies and conventional methods for detecting occlusal caries in permanent teeth. Brazilian Journal of Oral Sciences.27-32.

Rocha RO, Ardenghi TM, Oliveira LB, Rodrigues CR, Ciamponi AL. 2003. In vivo effectiveness of laser fluorescence compared to visual inspection and radiography for the detection of occlusal caries in primary teeth. Caries research. 37(6):437-441.

Rodrigues J, A., Hug I, Diniz M, Lussi A. 2008. Performance of fluorescence methods, radiographic examination and icdas ii on occlusal surfaces in vitro. Caries research. 42(4):297-304.

Rodrigues JA, Diniz MB, Josgrilberg EB, Cordeiro RC. 2009. In vitro comparison of laser fluorescence performance with visual examination for detection of occlusal caries in permanent and primary molars. Lasers in medical science. 24(4):501-506.

Seremidi K, Lagouvardos P, Kavvadia K. 2012. Comparative in vitro validation of vistaproof and diagnodent pen for occlusal caries detection in permanent teeth. Operative dentistry. 37(3):234-245.

Shi XQ, Welander U, Angmar-Mansson B. 2000. Occlusal caries detection with kavo diagnodent and radiography: An in vitro comparison. . Caries Research 34:151-158.

Shimada Y, Nakagawa H, Sadr A, Wada I, Nakajima M, Nikaido T, et a. 2014. Noninvasive cross-sectional imaging of proximal caries using swept-source optical coherence tomography (ss-oct) in vivo. Journal of biophotonics. 7(7):506-513.

Shimada Y, Sadr A, Burrow M F, Tagami J, Ozawa N, Sumi Y. 2010. Validation of swept-source optical coherence tomography (ss-oct) for the diagnosis of occlusal caries. Journal of dentistry. 38:655-665.

Sidi AD, Naylor MN. 1988. A comparison of bitewing radiography and interdental transillumination as adjuncts to the clinical identification of approximal caries in posterior teeth. British dental journal. 164:15-18.

Simon JC, Lucas SA, Staninec M, Tom H, Chan K H, Darling CL, et a. 2016. Near-ir transillumination and reflectance imaging at 1,300nm and 1,500-1,700nm for in vivo caries detection. Lasers in surgery and medicine. 48(9):828-836.

Souza J, Boldieri T, Diniz M B, Rodrigues J, Lussi A, Cordeiro R. 2013. Traditional and novel methods for occlusal caries detection: Performance on primary teeth. Lasers in medical science. 28(1):287-295.

Soviero VM, Leal SC, Silva RC, Azevedo RB. 2012. Validity of microct for in vitro detection of proximal carious lesions in primary molars. Journal of dentistry. 40(1):35-40.

Sridhar N, Tandon S, Rao N. 2009. A comparative evaluation of diagnodent with visual and radiography for detection of occlusal caries: An in vitro study. Indian journal of dental research. 20(3):326-331.

Teo TK, Ashley PF, Louca C. 2014. An in vivo and in vitro investigation of the use of icdas, diagnodent pen and cariescan pro for the detection and assessment of occlusal caries in primary molar teeth. Clinical oral investigations. 18(3):737-744.

Tonkaboni A, Saffarpour A, Aghapourzangeneh F, Fard MJK. 2018. Comparison of diagnostic effects of infrared imaging and bitewing radiography in proximal caries of permanent teeth. Lasers in medical science.

Van Hilsen Z, Jones R. 2013. Comparing potential early caries assessment methods for teledentistry. BMC oral health. 13:16-16.

Virajsilp V, Thearmontree A, Aryatawong S, Paiboonwarachat D. 2005. Comparison of proximal caries detection in primary teeth between laser fluorescence and bitewing radiography. Pediatric dentistry. 27(6):493-499.

Xiao-Hua D, Hui Y, Xiaoli L, Yanni L, Yingying W, Xiaobin L, et a. 2016. Ex vivo assessment of the potency of optical coherence tomography for the detection of early occlusal caries. Hua xi kou qiang yi xue za zhi [West China journal of stomatology]. 34:564-569.
